# Effectiveness of acupuncture combined with rehabilitation training vs. rehabilitation training alone for post-stroke shoulder pain: A systematic review and meta-analysis of randomized controlled trials

**DOI:** 10.3389/fmed.2022.947285

**Published:** 2022-10-04

**Authors:** Jie Zhan, Xiaojing Wei, Chenyang Tao, Xiaoting Yan, Peiming Zhang, Rouhao Chen, Yu Dong, Hongxia Chen, Jianhua Liu, Liming Lu

**Affiliations:** ^1^Postdoctoral Research Station, Department of Rehabilitation, Guangdong Provincial Hospital of Chinese Medicine, The Second Affiliated Hospital of Guangzhou University of Chinese Medicine, Guangzhou, China; ^2^Clinical Research and Data Center, South China Research Center for Acupuncture and Moxibustion, Medical College of Acu-Moxi and Rehabilitation, Guangzhou University of Chinese Medicine, Guangzhou, China; ^3^Department of Rehabilitation, Guangdong Provincial Hospital of Chinese Medicine, Guangzhou, China; ^4^Research Team for Acupuncture Effect and Mechanism, The Second Affiliated Hospital of Guangzhou University of Chinese Medicine, Guangzhou, China

**Keywords:** post-stroke shoulder pain, acupuncture, rehabilitation training, alternative and complementary medicine, meta-analysis

## Abstract

**Background:**

Post-stroke shoulder pain (PSSP) is characterized by shoulder pain on the hemiplegic side, which can limit physical activity in patients with stroke. Acupuncture combined with rehabilitation training (AR) has been widely used in PSSP, but the evidence of its effectiveness is still unclear.

**Objective:**

The study aimed to evaluate the effect and safety of AR vs. rehabilitation training (RT) alone on PSSP.

**Methods:**

We searched PubMed, the Cochrane Library, the Chinese Biological Medicine Database (CBM), the Chinese Scientific Journal Database (VIP), China National Knowledge Infrastructure (CNKI), and the WAN FANG database for relevant studies from their inception to February 2022. Only randomized controlled trials (RCTs) comparing the effect of AR with RT alone on PSSP were considered. The primary outcome was shoulder pain. Secondary outcomes included upper limb motor function, activities of daily living (ADL), shoulder range of motion (ROM), and adverse events (AEs). Subgroup analysis and sensitivity analysis were also conducted. Quality assessment was implemented based on Cochrane risk of bias (ROB) criteria, which consist of seven items. When more than four items in a study were judged as low ROB, the overall quality of this study was considered low risk.

**Results:**

A total of 40 studies were included in the qualitative analysis, and 35 (87.5%) studies with 2,554 patients were included in the meta-analysis. Of the 40 studies, 14 (35.0%) were of moderate-to-high quality. The meta-analysis results showed that AR is better than RT alone in reducing shoulder pain (MD −1.32, 95% CI −1.58 to −1.07), improving upper limb motor function (MD 6.81, 95% CI 4.95–8.67), ADL (MD 11.17, 95% CI 9.44–12.91), and shoulder ROM (internal rotation: MD 10.48, 95% CI 8.14–12.83; backward extension: MD 7.82, 95% CI 6.00–9.64; anteflexion: MD 12.88, 95% CI 5.47–20.29; external rotation: MD 11.40, 95% CI 6.17–16.64; abduction: MD 16.96, 95% CI 8.61–25.31) without obvious AEs.

**Conclusion:**

AR may be better than RT alone for the improvement of shoulder pain, upper limb motor function, ADL, and shoulder ROM, without obvious AEs in patients with PSSP. However, considering the clinical and statistical heterogeneity, our findings need to be interpreted with caution. More rigorous RCTs in this area should be conducted in the future.

**Systematic review registration:**

[www.crd.york.ac.uk], identifier [CRD42022326763].

## Introduction

Post-stroke shoulder pain (PSSP) is a common complication of stroke, characterized by shoulder pain on the hemiplegic side (1–3). PSSP occurs more frequently in the chronic phase after stroke than in the acute phase and usually occurs 2–3 months after stroke (4). Recently, a review including 3,496 patients with stroke demonstrated that the relatively conservative estimate of the total annual incidence of PSSP fluctuates at 0.30 (1). The prevalence may be higher among those in rehabilitation because they tend to have more risk factors (5). To date, due to the etiology of shoulder pain being complex and multifactorial, the exact pathogenesis of PSSP still remains controversial (6, 7). Altered peripheral and central nervous activities, such as spasticity, severe arm paralysis, central post-stroke pain, complex regional pain syndrome, and central hypersensitivity, are considered to be related to PSSP, as well as musculoskeletal disorders, such as supraspinatus tendon pathology, frozen shoulder, and impingement syndrome (5, 8–11). Furthermore, the aforementioned causes may contribute to the development of PSSP individually or in combination. The negative effects of PSSP, such as limited physical activity, depressive states, and sleep disturbances, significantly deteriorate patients’ quality of life (12–15), predispose patients to withdraw from rehabilitation programs, and prolong hospitalization (1, 16), which impose a great burden on both patients and society.

Rehabilitation training (RT) and symptomatic treatment (physical therapy, occupational therapy, transcutaneous electrical nerve stimulation, peripheral nerve stimulation, robotic-assisted shoulder rehabilitation, good limb position, and ROM exercises for the affected shoulder) are the main intervention methods of PSSP (7, 17–20). However, shortcomings exist in the single rehabilitation treatment, including the short duration of efficacy, the limited scope of medication indications, potential adverse drug reactions, and most importantly, limited analgesic efficacy (21–23). For example, neuromuscular electrical stimulation has preventive and therapeutic effects on subluxation, but not pain relief (24, 25). Pharmacological therapy such as corticosteroid injections may not be applied to some type of PSSP owing to its potential side effects (26).

In recent years, acupuncture has become an increasingly popular technique and is used worldwide for the management of pain, headache, musculoskeletal diseases, and other health problems (27). Previous studies demonstrated that acupuncture is effective and safe for PSSP, especially in reducing pain intensity (27–30). It has been incorporated into some guidelines as adjuvant therapy for PSSP (31). Acupuncture combined with rehabilitation (AR) is more effective than rehabilitation training (RT) alone in relieving shoulder pain, improving upper limb movement, and increasing joint range of motion (ROM) in patients with PSSP (17, 32–34). The combined therapy can also shorten the treatment duration, increase blood flow, and reduce edema with fewer adverse reactions (32, 35–37). In 2015, a review including 13 randomized controlled trials (RCTs) demonstrated that AR may be better than RT alone in reducing pain and improving upper limb motor function and activities of daily living (ADL) (38). The number of studies included in this meta-analysis was limited, the quality of studies included was mostly low, and the reliability of its conclusions needs to be improved. In recent years, a large number of RCTs focusing on AR in the treatment of PSSP have been published. We conducted this systematic review (SR) and meta-analysis (MA) to comprehensively update the existing evidence to clarify the role of AR in PSSP. The results of this study may provide evidence for the rehabilitation management of PSSP.

## Methods

We conducted this SR and MA according to the Preferred Reporting Items for Systematic Reviews and Meta-Analyses (PRISMA). Only RCTs compared the efficacy of AR with RT alone on PSSP and published in Chinese or English were considered eligible for our MA. A total of six databases, such as PubMed and the Cochrane Library, were searched systematically from their inception to February 2022 for eligible studies. The protocol was registered in the International Prospective Register of Systematic Reviews (No. CRD42022326763). In this review, no ethical approval or patient consent was required because all data analyses were from previously published studies.

### Inclusion and exclusion criteria

#### Types of studies

We included all RCTs comparing the effectiveness of AR with RT alone for PSSP. The studies should be published in Chinese or English. We contacted the authors by email if there was any incomplete information, and studies were excluded if data remained incomplete. The studies would still be excluded if (1) the study compared the effect of different RT techniques, different acupuncture parameters, or different types of acupuncture; and (2) the study type is crossover trials, case reports, animal experiments, abstracts, thesis, or review articles.

### Population

We included studies in which participants were diagnosed with ischemic or hemorrhagic stroke by recognized criteria or brain imaging technology (e.g., brain CT, MRI, and DSA). Studies were included if the participants in the study had hemiplegic shoulder pain following a stroke, and shoulder pain was assessed by using a visual analog scale (VAS), or other recognized instruments [e.g., McGi pain questionnaire (MPQ) (39), numeric rating scale (NRS) (40), and faces pain scale (FPS) (41)]. The inclusion criteria included participants > 18 years, regardless of gender, race, clinical setting, or the time since stroke. We excluded studies that recruited participants with the shoulder-hand syndrome (i.e., complex regional pain syndrome) or shoulder dislocation. We also excluded studies that included participants who suffered from shoulder periarthritis, shoulder trauma, and other shoulder diseases before the stroke.

### Intervention and comparison

We included studies assessing the effectiveness of AR vs. RT alone on PSSP. The types of acupuncture included auricular acupuncture, abdominal acupuncture (AA), balancing acupuncture (BAA), body acupuncture (BA), carpus–ankle acupuncture (CAA), electroacupuncture (EA), fire acupuncture (FA), relaxing needling at meridian-muscle nodes (RNN), scalp acupuncture (SA), traditional acupuncture (TA), and warm acupuncture (WA), but not moxibustion. The RT mainly included good limb position, Bobath, Brunnstrom, Rood, and shoulder joint ROM exercise. The participants in the AR group received AR, while the participants in the RT group, received RT alone. The RT regimens and base medicines in the two groups must be similar.

### Outcomes

#### Primary outcome

The primary outcome was shoulder pain assessed by VAS, NRS, MPQ, or FPS at the end of treatment. The VAS score changes between 0 and 10 points, the NRS uses the numbers 0–10 to indicate pain intensity, the MPQ evaluates pain *via* 11 questions, and the FPS uses different facial expressions to show six levels of pain. These scales indicate that the higher the score, the more severe the pain.

#### Secondary outcome

The secondary outcomes were the motor function of the upper limb, ADL, shoulder ROM, and adverse events (AEs). The assessment time of the outcomes was at the end of treatment. The upper limb motor function was assessed by the Fugl-Meyer scale for the upper limb (FMA-U), ADL evaluated by the Barthel Index (BI) or the modified Barthel Index (MBI), and shoulder ROM measured by the protractor. The FMA-U scale contains 33 items, with a full score of 66 points. The higher the FMA-U score, the better the upper limb function (42). The BI or MBI includes 10 items (e.g., eating, personal hygiene, bathing, toileting, dressing, anal control, bladder control, bed and chair transfer, level walking, and stairs), with a full score of 100 points. The lower the score, the more serious the ADL (43, 44). The potential AEs related to RT or AR may include local subcutaneous ecchymosis, nausea, dizziness, infection, and palpitation.

#### Data sources and searches

We searched six electronic databases from their inception to February 2022: PubMed, the Cochrane Library, the Chinese Biological Medicine Database (CBM), the Chinese Scientific Journal Database (VIP), China National Knowledge Infrastructure (CNKI), and the WAN FANG database. We performed a systematic search using Medical Subject Headings, titles, keywords, and free words related to acupuncture, RT, and PSSP. The detailed electronic search strategies for all databases are provided in Supplementary Appendix 1. We manually searched additional studies by screening the reference lists of the included articles and the relevant reviews.

### Data collection and analysis

#### Selection of studies

Initially, all articles were imported into EndNote (version X9) for automatic deduplication. After removing duplicates, two authors (XW and CT) independently reviewed the titles and abstracts of all articles to determine whether these articles met the inclusion and exclusion criteria. Full-text articles that potentially met the eligibility criteria were retrieved. Then, the same two authors independently read these full-text articles to identify eligible studies. When multiple studies described the same trial, we included only the earliest published study. During the study selection process, any disagreements could be resolved by discussion or by consulting with a third author (JZ) as necessary.

#### Data extraction and management

Microsoft Excel was used by two authors (XY and PZ) independently to extract predefined data from the studies included. Where a study considered multiple intervention groups, data were extracted only for AR and RT groups. These authors conducted data extraction in duplicate and then checked the accuracy of these data. During the data extraction process, any disagreements could be resolved by discussion or by consulting with a third author (JZ), as necessary. The predefined data included (1) basic characteristics of the study, such as author name, publication year, sample size, gender, age, time since stroke, and type of stroke; (2) detailed information about interventions, such as types of acupuncture, selected acupoints, duration of needle retention, and frequency and duration of treatment; and (3) outcome measures, such as VAS, FMA-U, BI/MBI, shoulder ROM, and AEs.

#### Quality assessment

The risk of bias (ROB) assessment tool in the *Cochrane Handbook for Systematic Reviews of Interventions* (45) was used by two authors (CT and XY) independently to assess the methodological quality of the studies included. This tool included seven criteria: randomization method, allocation concealment, blinding of the participant and personnel, blinding of outcome assessment, incomplete outcome data, selective reporting, and other bias. Each of the criteria is classified as “low risk,” “high risk,” or “unclear risk.” Where a study had more than four low risks in the seven criteria, we considered the overall quality of this study as moderate-to-high quality. Any disagreements could be resolved by discussion or by consulting with a third author (JZ), as necessary.

#### Measures of treatment effect

We used the post-treatment mean and standard deviation of the two groups to obtain the pooled effect size. When outcomes were evaluated by using the same scale, we used weighted mean differences (WMDs) with 95% confidence intervals (CIs) to describe continuous variables; otherwise, we used standardized mean differences (SMDs) with 95% CIs.

#### Unit of analysis issues

We managed and analyzed the data of non-standard design studies in accordance with the guidelines recommended in the Cochrane Handbook for Systematic Reviews of Interventions (version 5.1.0) (45).

#### Dealing with missing data

We contacted the corresponding authors or relevant authors of the studies included by email to obtain relevant data and information missing from the study. When the authors of these studies had provided data on at least one outcome among shoulder pain, upper limb motor function, and ADL before and after the intervention, we included those studies in our meta-analysis; otherwise, only a qualitative synthesis of those was performed.

#### Assessment of heterogeneity

We tested the clinical heterogeneity of studies included *via* analysis of the basic characteristics of participants (e.g., gender, age, type of stroke, and time since stroke), protocols of intervention (e.g., types of acupuncture, duration of needle retention, and frequency and duration of treatment), outcome measures, and trial design (e.g., randomization method, allocation concealment, and double-blinding).

We tested the statistical heterogeneity of the studies included using the Cochrane I-squared statistic. The I-squared statistic quantified the percentage of heterogeneity in the outcome measures. When I-squared was more than 25, 50, and 75%, the heterogeneity between studies was considered low, moderate, or high, respectively.

#### Assessment of reporting biases

We tested the publication biases by using visual funnel plots and Egger’s test when the number of studies included was beyond 10.

### Data synthesis

We conducted statistical analyses using RevMan version 5.3 (Nordic Cochrane Centre, the Cochrane Collaboration 2014) and RStudio Desktop 1.4.1717.^1^ Shoulder pain was the primary outcome of this meta-analysis. Given the small number of studies included and moderate-to-high heterogeneity, we conducted a meta-analysis for the primary outcome using a random effects model. For secondary outcomes (e.g., FMA-U, MBI, and ROM), we used a random effects model to pool data when the statistic heterogeneity was significant; otherwise, we used a fixed effects model to conduct the meta-analysis. Furthermore, we performed a narrative summary for AEs. For all meta-analyses, we considered two-tailed *P*-values less than 0.05 as statistically significant.

### Subgroup analysis

Considering the clinical heterogeneity of studies included, we performed subgroup analyses based on different types of acupuncture as follows: EA plus RT vs. RT alone, BAA plus RT vs. RT alone, and TA plus RT vs. RT alone.

### Sensitivity analysis

We conducted a sensitivity analysis for the primary outcome by removing each study individually to test the robustness of the meta-analysis results.

## Results

### Study selection

**FIGURE 1:**
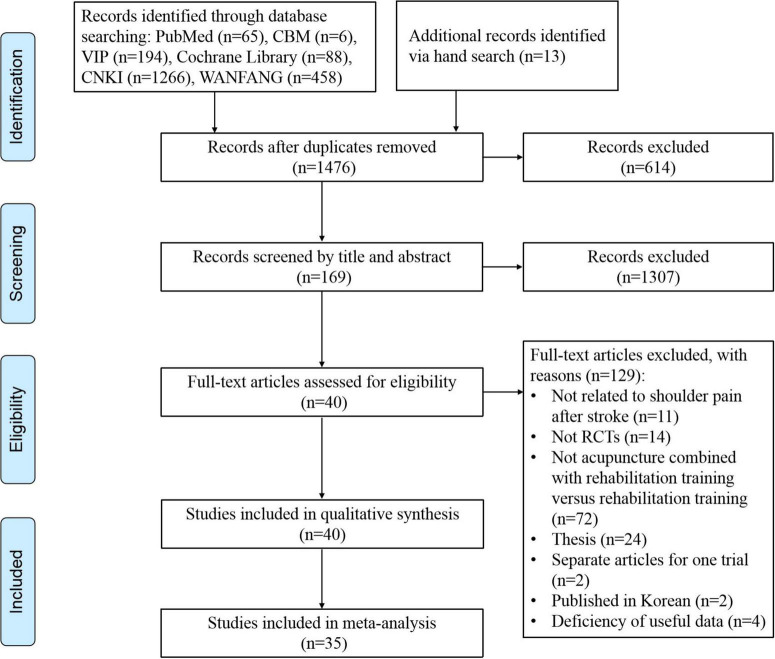
Study flow diagram.

In total, 2,090 electronic publications were identified from the selected databases. After removing duplicates (*n* = 614), we further excluded 1,307 articles by screening titles and abstracts, and then 169 articles were retained with full text. Of the 169 articles, 127 were removed for the following reasons: unrelated to PSSP (*n* = 11), not RCTs (*n* = 14), irrelevant intervention or comparison (*n* = 72), thesis (*n* = 24), separate articles for the same trial (*n* = 2), not published in Chinese or English (*n* = 2), and missing data (*n* = 2). Finally, 40 studies (46–85) were included in the qualitative analysis, and 35 studies with 2,554 patients were included in the meta-analysis due to five studies (54, 58, 60, 65, 77) without appropriate outcomes data. The selection flow of studies is described in Figure 1.

### Characteristics of eligible studies

With sample sizes ranging from 26 to 164, the publication years of studies included were distributed between 2002 and 2022. Except for 11 studies without reporting how long after stroke PSSP occurred, the remaining studies reported PSSP occurred in the acute and chronic phases of stroke, ranging from days to months. Overall, 20 studies covered stroke patients with infarction or hemorrhage, two studies (60, 84) were limited to cerebral infarction, and 18 studies had no further information on the type of stroke. Of the 40 studies included, 10 compared EA plus RT vs. RT alone, nine compared TA plus RT with RT alone, two compared AA plus RT vs. RT alone, three compared BAA plus RT with RT alone, four compared CAA plus RT vs. RT alone, two compared WA plus RT with RT alone, and four compared SA and BA plus RT vs. RT alone. The remaining studies compared the effectiveness of TA and BAA plus RT vs. RT alone, EA and BA plus RT vs. RT alone, SA plus RT vs. RT alone, WA and EA plus RT vs. RT alone, FA plus RT vs. RT alone, and RNN plus RT vs. RT alone, respectively. The highest needle retention time was 30 min, except for the fire acupuncture, which only needed 30 s. The treatment duration ranged from 5 to 60 sessions with the days ranging from 7 days to 4 months. Of the 40 eligible studies, 31 assessed shoulder pain by using VAS, one by NRS, one by FPS, and one by MPQ. A total of 29 studies assessed the upper limb motor function *via* the FMA-U, 12 studies used the MBI or BI to evaluate ADL, and five studies evaluated the shoulder ROM by the protractor. The characteristics of the included studies are given in Supplementary Table 1.

### The quality of studies included

**FIGURE 2:**
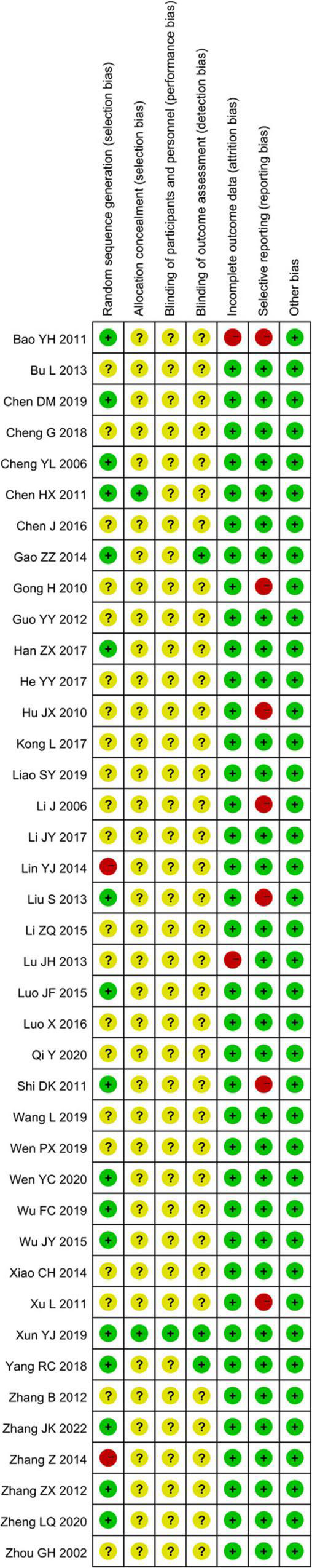
Risk of bias assessments of included studies.

Of the 40 eligible studies, 14 (35.0%) were of moderate-to-high quality based on the ROB criteria. In terms of selection bias, 17 articles (42.5%) reported on the randomization process using a random number table approach were judged as low risk, two were considered high risk due to inappropriate randomization procedures, and 21 were considered an unclear risk due to lack of reporting. As for allocation concealment, only two (5%) articles were classified as low risk because correct allocation concealment processes using opaque envelopes were reported. Others were judged as an unclear risk due to the absence of the random number allocation process. For performance bias, patients could not be blinded because there were no sham or placebo controls, so we focused on whether the articles had blinding of acupuncture practitioners. Of all, only one RCT (2.5%) was considered low risk because its acupuncturists were blinded to the grouping. In terms of the blinding of outcome assessment, only three articles (7.5%) were judged as low risk and their outcomes were assessed by those who did not participate in acupuncture practice and unknown to the grouping. Two studies (5%) reported cases of dropouts but did not specify the reasons for dropout, and 38 studies (95%), which reported all outcome data, were considered as low ROB due to attrition bias. In total, seven studies (17.5%) were judged as high risk of reporting bias because one or more outcomes of interest to this SR were poorly or not reported in these studies, which prevented them from being included in the meta-analysis. Other biases were not found in all the studies. The risk of bias assessments of studies included is shown in Figure 2.

### Meta-analysis

#### Primary outcome

##### Shoulder pain

**FIGURE 3:**
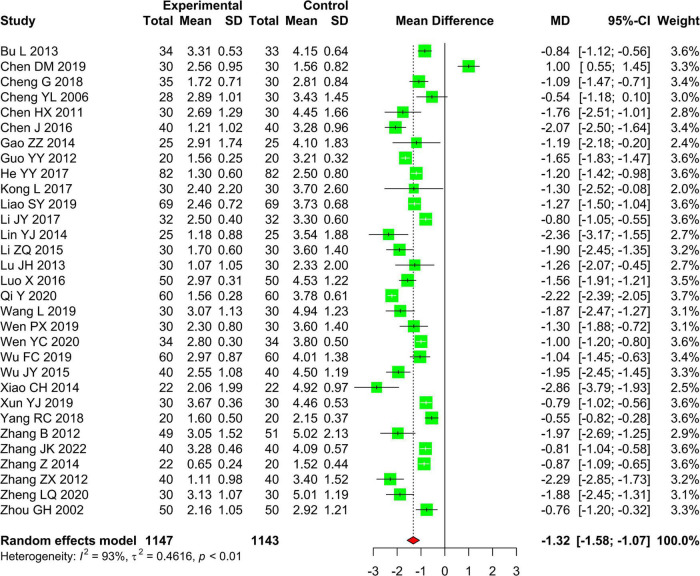
Forest plot and meta-analysis of VAS.

A total of 31 studies with 2,290 patients compared the effectiveness of AR vs. RT alone on shoulder pain assessed by using VAS. We used the random effects model to pool the data due to the high statistical heterogeneity (*I*^2^ = 93%, *P* < 0.01). The results of pooling data showed that AR is superior to RT alone in reducing shoulder pain in patients with PSSP (MD −1.32, 95% CI: −1.58 to −1.07, *Z* = −10.10, *P* < 0.01) (Figure 3). Only one study with 88 patients used the NRS to evaluate shoulder pain, and there was a significant difference between AR and RT alone (2.83 ± 2.24 vs. 3.95 ± 2.31, *P* < 0.05); one study with 60 patients used the FPS to assess the improvement of shoulder pain between AR and RT alone, and the difference between AR vs. RT alone was significant (1.23 ± 0.77 vs. 5.30 ± 1.44, *P* < 0.05); and one study including 25 patients used the MPQ to evaluate shoulder pain, and AR was better than RT alone in reducing shoulder pain (10.54 ± 3.01 vs. 17.25 ± 2.77, *P* < 0.05).

#### Secondary outcome

##### Motor function of upper limb

**FIGURE 4:**
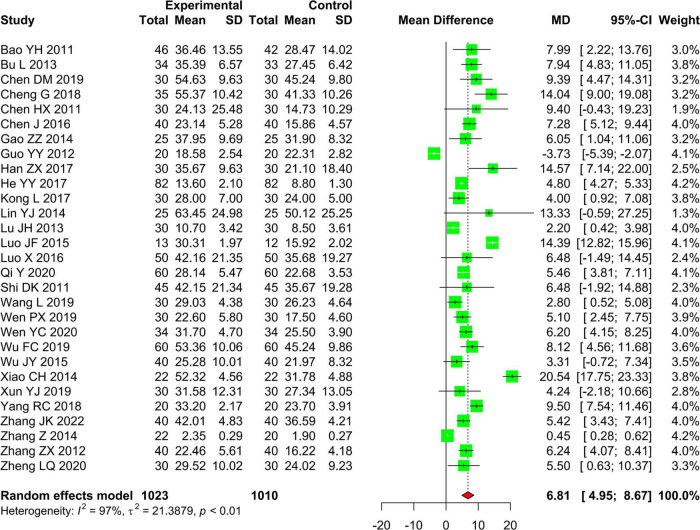
Forest plot and meta-analysis of FMA-U.

A total of 29 studies including 2,033 patients compared the effectiveness of AR vs. RT alone on upper limb motor function assessed by using the FMA-U. Because of significantly statistical heterogeneity (*I*^2^ = 97.3%%, *P* < 0.01), we selected the random effects model to pool the data. The results of the meta-analysis showed that AR is better than RT alone in improving the upper limb motor function (MD 6.81, 95% CI: 4.95–8.67, *Z* = 7.18, *P* < 0.01) (Figure 4).

##### Activities of daily living

**FIGURE 5:**
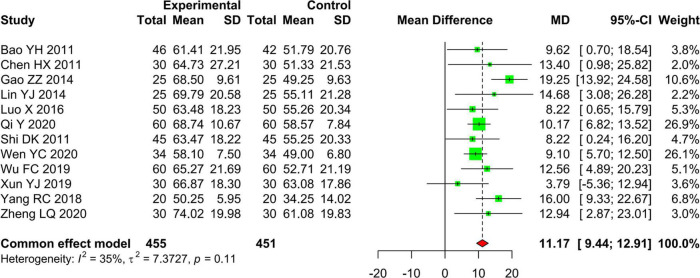
Forest plot and meta-analysis of MBI.

In total, 12 studies including 906 patients compared the effectiveness of AR vs. RT alone on ADL assessed by using the MBI or BI. We used the fixed effects model to pool the data because of the low statistical heterogeneity (*I*^2^ = 35.4%, *P* = 0.11). The results of pooling data showed that the effectiveness of AR on ADL is better than that of RT alone (MD 11.17, 95% CI: 9.44–12.91, *Z* = 12.61, *P* < 0.01) (Figure 5).

##### Shoulder range of motion

**FIGURE 6:**
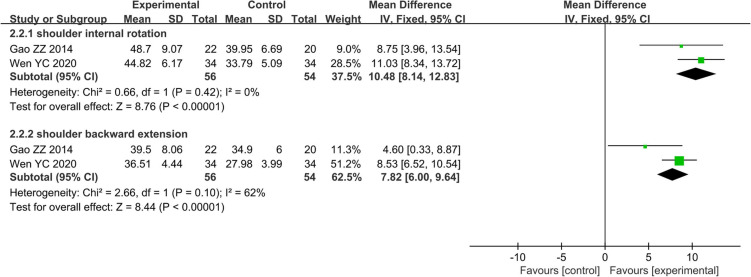
Forest plot and meta-analysis of ROM of internal rotation and backward extension.

Only two studies including 110 patients compared the effectiveness of AR vs. RT alone on ROM of shoulder internal rotation and backward extension, respectively. The effects on ROM of shoulder internal rotation and backward extension were analyzed by a fixed effects model due to low to moderate statistical heterogeneity (*I*^2^ = 0.0%, *P* = 0.42; *I*^2^ = 62.4%, *P* = 0.10; respectively). AR for the improvement of ROM of shoulder internal rotation and backward extension was better than RT alone, respectively (MD 10.48, 95% CI: 8.14–12.83, *Z* = 8.76, *P* < 0.01; MD 7.82, 95% CI: 6.00–9.64, *Z* = 8.44, *P* < 0.01; respectively) (Figure 6).

**FIGURE 7:**
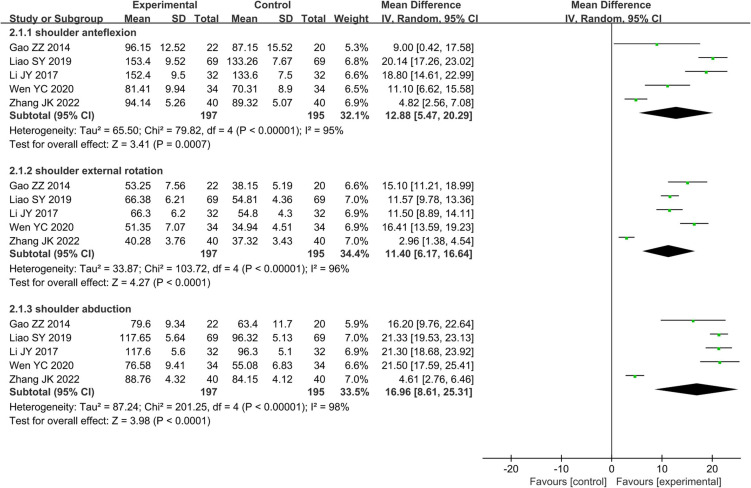
Forest plot and meta-analysis of ROM of anteflexion, external rotation, and abduction.

Only five studies including 392 patients compared the effectiveness of AR vs. RT alone on ROM of shoulder anteflexion, external rotation, and abduction, respectively. The effects on ROM of shoulder anteflexion, external rotation, and abduction was analyzed using a random effects model, owing to significant heterogeneity (*I*^2^ = 95.0%, *P* < 0.01; *I*^2^ = 96.0%, *P* < 0.01; *I*^2^ = 98.0%, *P* < 0.01; respectively). The effect of AR on ROM of shoulder anteflexion, external rotation, and abduction, respectively, was better than that of RT alone (MD 12.88, 95% CI: 5.47–20.29, *Z* = 3.41, *P* < 0.01; MD 11.40, 95% CI: 6.17–16.64, *Z* = 4.27, *P* < 0.01; MD 16.96, 95% CI: 8.61–25.31, *Z* = 3.98, *P* < 0.01; respectively) (Figure 7).

##### Adverse events

Only one study reported no AEs related to AR or RT alone, and the other studies did not mention AEs.

#### Subgroup analysis

##### Electroacupuncture plus rehabilitation training vs. rehabilitation training alone

**FIGURE 8:**
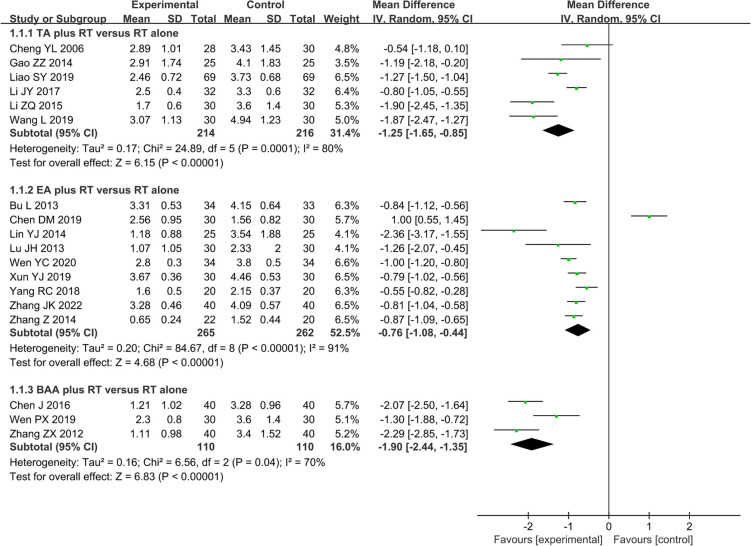
Forest plot and subgroup meta-analysis of VAS (BAA, balancing acupuncture; EA, electroacupuncture; RT, rehabilitation training; TA, traditional acupuncture; VAS, visual analog scale).

A total of nine studies including 527 patients compared the effectiveness of EA plus RT vs. RT alone on shoulder pain assessed by VAS. A random effects model was used to pool the data due to significant heterogeneity (*I*^2^ = 91%, *P* < 0.01). The results of the meta-analysis showed that EA plus RT was better than that RT alone in reducing shoulder pain (MD −0.76, 95% CI −1.08 to −0.44, *Z* = 4.68, *P* < 0.01) (Figure 8).

##### Traditional acupuncture plus rehabilitation training vs. rehabilitation training

Overall, six studies including 430 patients used VAS to assess shoulder pain. We used a random effects model to conduct meta-analysis because of significant heterogeneity (*I*^2^ = 80%, *P* < 0.01). There was a significant difference in reducing shoulder pain between TA plus RT and RT alone (MD −1.25, 95% CI −1.65 to −0.85, *Z* = 6.15, *P* < 0.01) (Figure 8).

##### Balancing acupuncture plus rehabilitation training vs. rehabilitation training alone

A total of three studies with 220 patients compared the effectiveness of BAA plus RT vs. RT alone on shoulder pain assessed by VAS. Due to a significant heterogeneity (*I*^2^ = 70%, *P* = 0.04), we used a random effects model to pool the data. BAA plus RT was better than RT alone in reducing shoulder pain (MD −1.90, 95% CI −2.44 to −1.35, *Z* = 6.83, *P* < 0.01) (Figure 8).

#### Sensitivity analysis

The stability of the pool data of the primary outcome was tested by removing studies one by one, and the result also supports that the effectiveness of AR in reducing shoulder pain was better than that of RT alone (*P* < 0.01) (Table 1).

**TABLE 1:** Results of sensitivity analysis based on VAS.

	MD	95%-CI	*P*-value	tau^2^	tau	*I* ^2^
Omitting Bu L 2013	−1.3412	[−1.6052; −1.0771]	<0.0001	0.4728	0.6876	93.0%
Omitting Chen DM 2019	−1.3888	[−1.5989; −1.1786]	<0.0001	0.2790	0.5282	91.1%
Omitting Cheng G 2018	−1.3320	[−1.5976; −1.0665]	<0.0001	0.4795	0.6925	93.1%
Omitting Cheng YL 2006	−1.3471	[−1.6076; −1.0867]	<0.0001	0.4613	0.6792	93.1%
Omitting Chen HX 2011	−1.3107	[−1.5735; −1.0478]	<0.0001	0.4719	0.6869	93.1%
Omitting Chen J 2016	−1.2966	[−1.5566; −1.0366]	<0.0001	0.4575	0.6764	92.9%
Omitting Gao ZZ 2014	−1.3268	[−1.5899; −1.0637]	<0.0001	0.4753	0.6894	93.1%
Omitting Guo YY 2012	−1.3113	[−1.5764; −1.0461]	<0.0001	0.4768	0.6905	92.8%
Omitting He YY 2017	−1.3285	[−1.5948; −1.0621]	<0.0001	0.4818	0.6942	93.1%
Omitting Kong L 2017	−1.3239	[−1.5860; −1.0617]	<0.0001	0.4733	0.6880	93.1%
Omitting Liao SY 2019	−1.3259	[−1.5923; −1.0594]	<0.0001	0.4822	0.6944	93.1%
Omitting Li JY 2017	−1.3427	[−1.6064; −1.0791]	<0.0001	0.4711	0.6863	92.9%
Omitting Lin YJ 2014	−1.2934	[−1.5503; −1.0365]	<0.0001	0.4488	0.6699	93.0%
Omitting Li ZQ 2015	−1.3041	[−1.5663; −1.0420]	<0.0001	0.4671	0.6835	93.0%
Omitting Lu JH 2013	−1.3254	[−1.5894; −1.0613]	<0.0001	0.4773	0.6908	93.1%
Omitting Luo X 2016	−1.3152	[−1.5806; −1.0498]	<0.0001	0.4787	0.6919	93.1%
Omitting Qi Y 2020	−1.2875	[−1.5442; −1.0309]	<0.0001	0.4425	0.6652	89.6%
Omitting Wang L 2019	−1.3057	[−1.5681; −1.0433]	<0.0001	0.4685	0.6845	93.1%
Omitting Wen PX 2019	−1.3245	[−1.5897; −1.0593]	<0.0001	0.4796	0.6925	93.1%
Omitting Wen YC 2020	−1.3358	[−1.6014; −1.0703]	<0.0001	0.4783	0.6916	93.0%
Omitting Wu FC 2019	−1.3336	[−1.5988; −1.0684]	<0.0001	0.4784	0.6917	93.1%
Omitting Wu JY 2015	−1.3018	[−1.5635; −1.0402]	<0.0001	0.4646	0.6816	93.0%
Omitting Xiao CH 2014	−1.2819	[−1.5324; −1.0314]	<0.0001	0.4249	0.6519	92.9%
Omitting Xun YJ 2019	−1.3432	[−1.6068; −1.0796]	<0.0001	0.4706	0.6860	92.9%
Omitting Yang RC 2018	−1.3512	[−1.6114; −1.0909]	<0.0001	0.4573	0.6763	92.7%
Omitting Zhang B 2012	−1.3039	[−1.5651; −1.0426]	<0.0001	0.4652	0.6820	93.1%
Omitting Zhang JK 2022	−1.3425	[−1.6063; −1.0787]	<0.0001	0.4715	0.6866	92.9%
Omitting Zhang Z 2014	−1.3404	[−1.6049; −1.0760]	<0.0001	0.4740	0.6885	92.9%
Omitting Zhang ZX 2012	−1.2905	[−1.5472; −1.0337]	<0.0001	0.4457	0.6676	92.9%
Omitting Zheng LQ 2020	−1.3051	[−1.5674; −1.0428]	<0.0001	0.4680	0.6841	93.1%
Omitting Zhou GH 2002	−1.3427	[−1.6057; −1.0797]	<0.0001	0.4697	0.6853	93.1%
Pooled estimate	−1.3228	[−1.5794; −1.0662]	<0.0001	0.4616	0.6794	92.90%

MD, mean difference; CI, confidence interval. Details on meta-analytical method: Inverse variance method; Restricted maximum-likelihood estimator for tau^2^.

#### Publication bias

**FIGURE 9:**
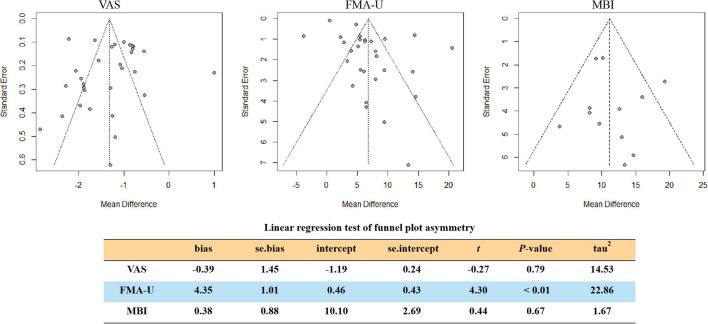
Funnel plots illustrating meta-analysis of VAS, FMA-U, and MBI. (FMA-U, Fugl-Meyer Assessment Scale for upper extremity; MBI, modified Barthel Index; VAS, visual analog scale).

All included studies were roughly well distributed on both sides of the funnel based on VAS and the MBI. Meanwhile, Egger’s test based on VAS (*t* = −0.27, *P* = 0.79) and the MBI (*t* = 0.44, *P* = 0.67) also did not find obvious publication bias. However, some studies based on the FMA-U did not distribute inside 95% CIs, and Egger’s test (*t* = 4.30, *P* < 0.01) demonstrated obvious publication bias (Figure 9).

## Discussion

Our systematic review contained 40 RCTs focusing on the effectiveness of AR vs. RT alone for the improvement of symptoms and function in PSSP. Of the 40 studies included, 14 (35.0%) were of moderate-to-high quality based on the ROB criteria. The meta-analysis results of 35 RCTs with 2554 patients demonstrated that AR is better than RT alone in reducing shoulder pain and improving upper limb motor function, ADL, and shoulder ROM, without obvious AEs. Nevertheless, these conclusions must be interpreted with caution on account of substantial heterogeneity between studies.

In this meta-analysis, we found AR was superior to RT alone in the improvement of shoulder pain, motor function of the upper limb, ADL, and shoulder ROM of patients with PSSP. The robustness of the meta-analysis results was also confirmed by sensitivity analysis. In addition, a subgroup analysis of the efficacy of acupuncture on analgesia was conducted based on the different acupuncture types. Efficacy data of the same type of acupuncture from a single study were excluded for a more reliable result. The pooled results revealed that the EA, or TA, or BAA plus RT was better than that of RT alone on shoulder pain in patients with PSSP. Beyond that, when RT was combined with BAA, patients experienced a greater reduction in shoulder pain than combined with TA or EA, as well as with relatively low heterogeneity. The BAA seems to be a more promising solution for PSSP among the varied acupuncture. This may be a helpful finding for future research on acupuncture for PSSP. Overall, the heterogeneous sources of studies included mainly were diversified acupuncture regimens, such as the selected acupoints, manipulation of acupuncture, needle retention duration, and frequency and session of acupuncture. For instance, among RCTs using TA as intervention, one study (52) reported that the acupuncturist performed neutral supplementation and the draining method after stabbing the needles, while another study (53) reported that the acupuncturist conducted the lifting—thrusting supplementation and draining method to achieve Deqi [i.e., a feeling of soreness, numbness, distension, heaviness, or the electric shock sensation (86)]. Only 35% of the included articles were rated as moderate-to-high quality, which reflects the high possibility of methodological heterogeneity in the included literature. As we can see, neither randomization nor blinding was performed for the majority of RCTs included.

From the perspective of traditional Chinese medicine, pain is caused by the blockage of the meridians and collaterals, and acupuncture uses metal needles to penetrate specific acupoints in the body to regulate the Qi in the meridians and achieve its analgesic effect by unblocking the meridians and collaterals (87–89). From the perspective of modern medicine, acupuncture analgesia is a comprehensive effect that is considered to be achieved by the transmission of impulse signals generated by acupoints to the nervous system, thereby adjusting the generation and release of neurotransmitters (88, 90, 91). Some neurotransmitters related to pain regulation pathways, such as opioid peptides, γ-aminobutyric acid (GABA), 5-hydroxytryptamine (5-HT), glutamate, and norepinephrine (92–95), have been found to be involved in acupuncture analgesia. The most well-recognized mechanism therein of acupuncture analgesia is the endogenous opioid mechanism (88, 90). However, the exact physiological mechanism of acupuncture analgesia is still unclear.

Compared with previous studies, three reviews are similar to ours, published in 2018 (27), 2015 (46), and 2012 (96). The review published in 2018 specified a wider range of shoulder pain for eligible patients, including shoulder-hand syndrome. Also, the review took routine stroke care as the comparative intervention, which may make the conclusion less specific. In addition, only narrative summaries, rather than meta-analyses of data, were performed in the review, which made the conclusions lack the support of objective data. The reviews published in 2015 and 2012 have different inclusion criteria for eligible studies, such as the study type, control group, outcomes measurements, and quality assessment. The quality of the literature included in the aforementioned reviews was generally low, and there was a high risk of selective bias and measurement bias. Moreover, these three reviews have been published for a long time, and there may be hysteresis in their conclusions. In addition, as shown in Supplementary Table 1, some evidence of RCTs for AR on PSSP has emerged since 2018. Therefore, a comprehensive update of the available evidence is necessary to clarify the role of AR in PSSP.

There were some advantages in this review. We performed a comprehensive literature search using a combination of machine and manual methods after consulting professional library searchers. Meanwhile, we also rigorously conducted this SR and MA in accordance with PRISMA and the guideline of Cochrane Collaboration. In addition, we performed subgroup analysis and sensitivity analysis on the included studies, further investigated the effects of different types of acupuncture therapy, and explored the possible sources of heterogeneity among the studies. This can help our conclusions be more reliable and helpful to the actual condition.

There are some limitations to our study. First, we did not limit the intervention to a specific type of RT, which may lead to the amplification of the meta-analysis results. Second, we restricted the published languages of studies to Chinese or English, but only one eligible study was published in English, and all studies included were conducted in the Chinese population, which may lead to linguistic and regional biases that are difficult to eliminate. Third, due to the lack of clear descriptions of randomization, blinding, and allocation concealment in the protocols of most studies included, we cannot judge whether the authors performed these steps, which may affect the accuracy of our findings. Finally, there was a significantly statistical heterogeneity in this meta-analysis, which may increase the uncertainty of our results.

## Conclusion

In this review, we found AR is better than RT alone for the improvement of shoulder pain, upper limb motor function, ADL, and shoulder ROM, without obvious AEs in patients with PSSP. However, considering the clinical and statistical heterogeneity, our findings need to be interpreted with caution. In the future, more rigorous and standardized trials on AR for PSSP should be conducted.

## Data availability statement

The original contributions presented in this study are included in the article/Supplementary material, further inquiries can be directed to the corresponding author/s.

## Author contributions

JZ, JL, and LL were responsible for the conception and design of this systematic review. JZ and XW drafted the manuscript. JL and LL revised the manuscript. JZ and LL designed the search strategies. JZ and PZ conducted the electronic search. XW, RC, and YD manually screened the reference lists of the included studies and all relevant reviews. XY and PZ extracted the data. CT and XY independently assessed the risk of bias. JZ, XW, and LL analyzed and interpreted the data. HC and JZ arbitrated any disagreements during the process of systematic review. All authors approved the submitted version of the manuscript.

## Footnotes

1
https://www.rstudio.com/products/rstudio/download/
