# Supplementary material for: Effectiveness of acupuncture combined with rehabilitation training vs. rehabilitation training alone for post-stroke shoulder pain: A systematic review and meta-analysis of randomized controlled trials
Source: Front Med (Lausanne). 2022 Oct 4;9:947285. doi: 10.3389/fmed.2022.947285 (PMC9578557; doi:10.3389/fmed.2022.947285)
Supplement: Supplementary file 1 [file Data_Sheet_1.pdf]

Appendix 1：search in databases

Cochrane Library

#1 MeSH descriptor: [Stroke] explode all trees and with qualifier(s): [diagnosis - DI]

#2 (stroke):ti,ab,kw OR (cerebrovascular accident):ti,ab,kw OR (brain vascular accident):ti,ab,kw OR (hemiplegia):ti,ab,kw OR (monoplegia):ti,ab,kw (Word variations have been searched)

#3 (apoplexy):ti,ab,kw (Word variations have been searched)

#4 MeSH descriptor: [Reflex Sympathetic Dystrophy] explode all trees and with qualifier(s): [diagnosis - DI]

#5 MeSH descriptor: [Shoulder Pain] explode all trees and with qualifier(s): [diagnosis - DI]

#6 (shoulder hand syndrome):ti,ab,kw OR (shoulder pain):ti,ab,kw OR (reflex sympathetic dystrophy):ti,ab,kw (Word variations have been searched)

#7 MeSH descriptor: [Acupuncture] explode all trees

#8 MeSH descriptor: [Moxibustion] explode all trees

#9 MeSH descriptor: [Electroacupuncture] explode all trees

#10 (acupuncture):ti,ab,kw OR (moxibustion):ti,ab,kw OR (electroacupuncture):ti,ab,kw OR (fire needle):ti,ab,kw (Word variations have been searched)

#11 #1 or #2 or #3

#12 #4 or #5 or #6

#13 #7 or #8 or #9 or #10

#14 #11 and #12 and #13 with Cochrane Library publication date to Feb 2022

PubMed

#1 "stroke"[MeSH Terms] OR "stroke"[All Fields] OR "strokes"[All Fields] OR "stroke s"[All Fields] OR ("stroke"[MeSH Terms] OR "stroke"[All Fields] OR ("cerebrovascular"[All Fields] AND "accident"[All Fields]) OR "cerebrovascular accident"[All Fields]) OR ("apoplexies"[All Fields] OR "stroke"[MeSH Terms] OR "stroke"[All Fields] OR "apoplexy"[All Fields]) OR ("stroke"[MeSH Terms] OR "stroke"[All Fields] OR ("brain"[All Fields] AND "vascular"[All Fields] AND "accident"[All Fields]) OR "brain vascular accident"[All Fields]) OR ("stroke"[MeSH Terms] OR "stroke"[All Fields] OR ("cerebrovascular"[All Fields] AND "stroke"[All Fields]) OR "cerebrovascular stroke"[All Fields]) OR ("hemiplegia"[MeSH Terms] OR "hemiplegia"[All Fields] OR "hemiplegias"[All Fields]) OR ("hemiplegia"[MeSH Terms] OR "hemiplegia"[All Fields] OR "monoplegia"[All Fields] OR "monoplegias"[All Fields])

#2 "reflex sympathetic dystrophy"[MeSH Terms] OR ("reflex"[All Fields] AND

"sympathetic"[All Fields] AND "dystrophy"[All Fields]) OR "reflex sympathetic dystrophy"[All Fields] OR ("shoulder"[All Fields] AND "hand"[All Fields] AND "syndrome"[All Fields]) OR "shoulder hand syndrome"[All Fields] OR ("shoulder pain"[MeSH Terms] OR ("shoulder"[All Fields] AND "pain"[All Fields]) OR "shoulder pain"[All Fields]) OR ("reflex sympathetic dystrophy"[MeSH Terms] OR ("reflex"[All Fields] AND "sympathetic"[All Fields] AND "dystrophy"[All Fields]) OR "reflex sympathetic dystrophy"[All Fields])

#3 "acupunctural"[All Fields] OR "acupuncture"[MeSH Terms] OR "acupuncture"[All Fields] OR "acupuncture therapy"[MeSH Terms] OR ("acupuncture"[All Fields] AND "therapy"[All Fields]) OR "acupuncture therapy"[All Fields] OR "acupuncture s"[All Fields] OR "acupunctured"[All Fields] OR "acupunctures"[All Fields] OR "acupuncturing"[All Fields] OR ("moxibustion"[MeSH Terms] OR "moxibustion"[All Fields]) OR ("electroacupuncture"[MeSH Terms] OR "electroacupuncture"[All Fields] OR "electroacupuncturing"[All Fields]) OR (("fires"[MeSH Terms] OR "fires"[All Fields] OR "fire"[All Fields]) AND ("needle s"[All Fields] OR "needled"[All Fields] OR "needles"[MeSH Terms] OR "needles"[All Fields] OR "needle"[All Fields] OR "needling"[All Fields] OR "needlings"[All Fields]))

#4 #1 AND #2 AND #3 AND (1000/1/1:2022/2/28[pdat])

CNKI

（（（（ 主题%= 中风+卒中+脑梗死+脑栓塞+脑出血+脑血管意外+脑血管障碍 or 题名%= 中风+卒中+脑梗死+脑栓塞+脑出血+脑血管意外+脑血管障碍 or title= xls(中风)+xls(卒中)+xls(脑梗死)+xls(脑栓塞)+xls(脑出血)+xls(脑血管意外)+xls(脑血管障碍) or v_subject= xls(中风)+xls(卒中)+xls(脑梗死)+xls(脑栓塞)+xls(脑出血)+xls(脑血管意外)+xls(脑血管障碍)）AND（ 主题%= 肩痛+肩手综合征 or 题名%= 肩痛+肩手综合征 or title= xls(肩痛)+xls(肩手综合征) or v_subject= xls(肩痛)+xls(肩手综合征)））AND（ 主题%= 针灸+针刺+电针+毫针+火针+温针 or 题名%= 针灸+针刺+电针+毫针+火针+温针 or title= xls(针灸)+xls(针刺)+xls(电针)+xls(毫针)+xls(火针)+xls(温针) or v_subject= xls(针灸)+xls(针刺)+xls(电针)+xls(毫针)+xls(火针)+xls(温针)））AND（ 主题%= 针灸+针刺+电针+毫针+火针+温针 or 题名%= 针灸+针刺+电针+毫针+火针+温针 or title= xls(针灸)+xls(针刺)+xls(电针)+xls(毫针)+xls(火针)+xls(温针) or v_subject= xls(针灸)+xls(针刺)+xls(电针)+xls(毫针)+xls(火针)+xls(温针)））AND（ 发表时间 Between('1915-01-01','2022-02-28')）)；检索范围：总库。

CBM

#1 ("中风"[加权:扩展] OR "脑卒中"[加权:扩展] OR "脑梗死"[加权:扩展] OR "脑栓塞"[加权:扩展] OR "脑出血"[加权:扩展] OR "脑血管障碍"[加权:扩展])

#2 ("肩痛"[加权:扩展] OR "肩手综合"[加权:扩展])

#3 ("针灸"[加权:扩展] OR "针刺"[加权:扩展] OR "电针"[加权:扩展] OR "毫针"[加权:扩展] OR "火针"[加权:扩展] OR "温针"[加权:扩展] OR "灸法"[加权:

扩展])

#4 ((#3) AND (#2) AND (#1)) AND (临床试验[文献类型] OR 随机对照试验[文献类型] OR 多中心研究[文献类型]) AND (人类[特征词]) AND -2022[日期]

VIP

(((((((((题名或关键词=中风 OR 题名或关键词=脑卒中) OR 题名或关键词=脑梗死) OR 题名或关键词=脑栓塞) OR 题名或关键词=脑出血) OR 题名或关键词=脑血管意外) OR 题名或关键词=脑血管障碍) AND (题名或关键词=肩痛 OR 题名或关键词=肩手综合症))) AND ((((((题名或关键词=针灸 OR 题名或关键词=针刺) OR 题名或关键词=电针) OR 题名或关键词=毫针) OR 题名或关键词=火针) OR 题名或关键词=温针) OR 题名或关键词=灸法)))

WAN FANG

((主题:(中风 or 脑卒中 or 脑梗死 or 脑栓塞 or 脑出血 or 脑血管障碍 or 脑血管意外) and 主题:(针灸 or 针刺 or 电针 or 毫针 or 火针 or 温针 or 灸法) and 主题:(肩痛 or 肩手综合症))) and Date:*-2022
