# Supplementary material for: Effectiveness of acupuncture combined with rehabilitation training vs. rehabilitation training alone for post-stroke shoulder pain: A systematic review and meta-analysis of randomized controlled trials
Source: Front Med (Lausanne). 2022 Oct 4;9:947285. doi: 10.3389/fmed.2022.947285 (PMC9578557; doi:10.3389/fmed.2022.947285)
Supplement: Supplementary file 2 [file Table_1.pdf]

Table 1 The characteristics of included studies

Study	Sample size	Time since stroke (m)	Age (mean±SD)	Sex (male/female)	Type of stroke (infarction/ hemorrhage)	Regimen	Treatment Duration	Needle retention duration	The selected acupoints	Main outcomes
Bao YH 2011	T: 46<br>C: 42	T: 2.63±1.42<br>C: 2.59±1.41	T: 67.39±9.75<br>C: 64.85±8.90	T: 23/23<br>C: 22/20	T: 34/12<br>C: 31/11	T: EA plus RT<br>C: RT	30 sessions, 1 m	30 min	SI9, SJ14, LI10, LI11, LI14, LI15, SJ5	NRS, FMA-U, BI
Bu L 2013	T: 34<br>C: 33	T: 2.62±1.46<br>C: 2.57±1.41	T: 64.69±9.65<br>C: 65.13±9.24	T: 17/17<br>C: 17/16	T: 22/12<br>C: 23/10	T: EA plus RT<br>C: RT	60 sessions, 2 m	20 min	LI11, LI15, SI9, Biliao acupoint, Jianqian acupoint	VAS, FMA-U
Chen DM 2019	T: 30<br>C: 30	T: 20.57±3.77 d<br>C: 20.43±4.26 d	T: 54.36±8.46<br>C: 55.27±9.70	T: 18/12<br>C: 19/11	T: 20/10<br>C: 22/8	T: EA plus RT<br>C: RT	18 sessions, 3 w	30 min	LI14, LI15, SI9, SJ14, Jianqian acupoint	VAS, FMA
Cheng G 2018	T:35<br>C:30	NR	T: 54.37±10.25<br>C: 54.05±10.13	T: 20/15<br>C: 17/13	T: 18/17<br>C: 16/14	T: TA and BAA plus RT<br>C: RT	1 m	TA: 20-30 min;<br>BAA: 3 min	BAA: BP-HN1, BP-LE6, BP-HN4;<br>TA: A-shi acupoint, LI15, SJ14, SI9, PC6, LU5, HT1	VAS, FMA
Cheng YL 2006	T:28<br>C:30	T: 2.98±1.23<br>C: 2.62±1.02	T: 59.2（27-74）<br>C: 57.8（29-73）	T: 18/10<br>C: 21/9	T: 21/7<br>C: 22/8	T: TA plus RT<br>C: RT	30 sessions, 1 m	30 min	conventional acupoints, LI4, ST36	VAS
Chen HX 2011	T: 30<br>C: 30	T: 40.21±10.93 d<br>C: 41.57±9.81 d	T: 64.12±13.28<br>C: 62.79±10.51	T: 17/13<br>C: 18/12	T: NR<br>C: NR	T: AA plus RT<br>C:RT	12 sessions, 2 w	30 min	RN12, KI17, ST26, Shangfengshi point, Shangfengshiwai point	VAS, FMA-U, MBI
Chen J 2016	T:40<br>C:40	44.31±2.37 d	62.35±2.17	43/47	T: NR<br>C: NR	T: BAA plus RT<br>C: RT	21 sessions, 3 w	10-20 min	BP-LE6	VAS, FMA-U
Gao ZZ 2014	T: 25<br>C: 25	T: 2.97±1.51<br>C: 2.75±1.43	T: 54.13±9.25<br>C: 52.97±9.15	T: 15/10<br>C: 13/12	T: 12/13<br>C: 13/12	T: TA plus RT<br>C: RT	24 sessions, 4 w	30 min	SI9, SI11,SJ5, SJ14, HT1, LI4, LI10, LI11, LI15, PC6	VAS, FMA, MBI
Gong H 2010	T: 30<br>C: 30	T: 2.83±1.39<br>C: 2.93±1.38	T: 54.74±7.19<br>C: 55.29±7.05	T: 11/19<br>C: 10/20	NR<br>NR	T: SA and BA plus RT<br>C: RT	24 sessions, 4 w	SA: 6 h;<br>BA: 30 min	SA: 2 acupoints zone (Ding zone, Dingqian zone)<br>BA: LI15, SI9, SJ13	NR
Guo YY 2012	T: 20<br>C: 20	T: 42.56±12.78 d<br>C: 38.35±9.46 d	T: 64.15±13.28<br>C: 62.79±10.51	T: 12/18<br>C: 13/7	NR<br>NR	T: WA plus RT<br>C: RT	20 sessions, 4 w	NR	LI15, Jianqian acupoint, Jianhou acupoint, Biyu acupoint, A-shi acupoint	VAS, FMA, FIM

Table 1 *continues 1*

Study	Sample size	Time since stroke (m)	Age (mean±SD)	Sex (male/female)	Type of stroke (infarction/ hemorrhage)	Regimen	Treatment Duration	Needle retention duration	The selected acupoints	Main outcomes
Han ZX 2017	T: 30<br>C: 30	NR<br>NR	T: 65±10<br>C: 65±9	T: 19/11<br>C: 14/16	NR<br>NR	T: RNN plus RT<br>C: RT	30 sessions, 6 w	30 min	meridian-muscle nodes	FPS, FMA, SS-QOL
He YY 2017	T: 82<br>C: 82	T: 59.6±16.2<br>C: 59.4±16.5	T: 68.7±5.1<br>C: 68.2±5.2	T: 44/38<br>C: 43/39	NR<br>NR	T: SA and BA plus RT<br>C: RT	60 sessions, 72 d	SA: 10 min; BA: 30 min	SA: vasomotor zone of the scalp acupoints, MS6, EX-HN1, DU20<br>BA:SI3, SJ5, PC6, LI4, LI11, LI15, HT3, HT1, KI3, GB39, GB34, SP6, SP9, ST32, BL37, BL60	VAS, FMA
Hu JX 2010	T: 40<br>C: 39	T: 40.3±21.5 d<br>C: 42.3±22.1 d	T: 63±7<br>C: 61±6	T: 25/15<br>C: 24/15	T: 35/5<br>C: 35/4	T: TA plus RT<br>C: RT	15 sessions, 3 w	30 min	LI4, LI10, LI11, LI14, LI15, SI9, SI11, SI3, A-shi acupoint	VAS[#], FMA-U[#]
Kong L 2017	T: 30<br>C: 30	beyond 6	T: 63<br>C: 64	T: 18/12<br>C: 19/11	NR<br>NR	T: AA plus RT<br>C: RT	20 sessions, 4 w	30 min	RN4, RN6, RN10, RN12, KI17, ST24, Sanjiao acupoint	VAS, FAM-U
Liao SY 2019	T: 69<br>C: 69	T: 7-38 d<br>C: 7-41 d	T: 65.4±9.2<br>C: 66.1±9.8	T: 38/31<br>C: 39/30	NR<br>NR	T: Acupuncture plus RT<br>C: RT	40 sessions, 8 w	20 - 30 min	Jianqian acupoint, GB21, LI11, LI15, SJ5, SI9, SI11, SI12, SJ14, A-shi acupoint	VAS, ROM
Li J 2006	T: 20<br>C: 20	NR<br>NR	NR<br>NR	NR<br>NR	T: 20/0<br>C: 20/0	T: TA plus RT<br>C: RT	28 sessions, 4 w	30 min	LI4, LI10, LI11, LI15, SI11, LI13,PC6, SJ5	NR
Li JY 2017	T: 32<br>C: 32	T: 3.80±1.40<br>C: 3.50±1.70	T: 62.80±7.10<br>C: 63.50±6.80	T: 13/19<br>C: 11/21	NR<br>NR	T: TA plus RT<br>C: RT	40 sessions, 8 w	20 - 30 min	Jianqian acupoint, GB21,LI15, SI9, SI11, SI12, SJ14, A-shi acupoint	VAS, ROM
Lin YJ 2014	T: 25<br>C: 25	NR	56.9±7.44	26/24	28/22	T: EA plus RT<br>C: RT	12 sessions, 2 w	30 min	SI9, SI11, SI13, SJ14, GB21, A-shi acupoint	VAS, FMA-U, MBI
Liu S 2013	T: 33<br>C: 27	T: 3.2±0.1<br>C: 3.0±0.2	T: 55.0±3.5<br>C: 53±4.9	T: 19/14<br>C: 18/9	NR<br>NR	T: SA and BA plus RT<br>C: RT	112 sessions, 4 m	30 min	BA: BL20, BL21, ST36, ST40, SP10, BL17;<br>SA: DU20, EX-HN1,MS6, vasomotor zone of the scalp acupoints	NR

Table 1 ***continues 2***

Study	Sample size	Time since stroke (m)	Age (mean±SD)	Sex (male/female)	Type of stroke (infarction/ hemorrhage)	Regimen	Treatment Duration	Needle retention duration	The selected acupoints	Main outcomes
Li ZQ 2015	T: 30<br>C: 30	T: 20-100 d<br>C: 21-98 d	T: 59.2±11.5<br>C: 60.2±11.4	T: 18/12<br>C: 19/11	NR<br>NR	T: TA plus RT<br>C: RT	30 sessions, 1 m	30 min	A-shi acupoint	VAS
Lu JH 2013	C: 30<br>T: 30	C: 1.57±0.88<br>T: 1.49±0.95	C: 61.63±11.13<br>T: 61.80±11.38	C: 25/5<br>T: 19/11	C:23/7<br>T:24/6	T: EA plus RT<br>C: RT	20 sessions, 1 m	30 min	LI4, LI14, LI15, LI16, GB21, SI9, SI10, SI11, SI13, SJ5, SJ14, GB22, GB23, A-shi acupoint, EX-B2, MS6	VAS, FMA
Luo JF 2015	T: 13<br>C: 13	T: 53.00±20.39 d<br>C: 51.31±19.86 d	T: 57.15±9.97<br>C: 57.69±8.62	T: 8/5<br>C: 9/4	T: 8/5<br>C: 9/4	T: TA plus RT<br>C: RT	30 sessions, 1 m	30 min	SI11, LI4, LI10, LI11, LI15, PC6, SJ5	MPQ, FMA-U
Luo X 2016	T: 50<br>C: 50	less than 8	64.2±3.8	57/43	NR	T: CAA plus RT<br>C: RT	5 sessions, 10 d	30 min	acupoints upper Four, Five and Six	VAS, FMA, BI
Qi Y 2020	T: 60<br>C: 60	T: 56.48±17.82 d<br>C: 56.28±6.45 d	T: 55.64±6.72<br>C: 56.28±6.45	T: 33/27<br>C: 31/29	NR	T: SA and BA plus RT<br>C: RT	20 sessions, 1 m	BA: 20 min;<br>SA: 10 min	BA: SI3, SI9, SI11, SJ3, SJ5, SJ14, LI5, LI14, LI15, GB21<br>SA: MS7, MS9	VAS, FMA-U, BI
Shi DK 2011	T: 45<br>C: 45	18	35-85	NR	NR	T: CAA plus RT<br>C: RT	10 sessions, 20 d	30 min	acupoints upper Four, Five and Six	VAS#, FMA, BI
Wang L 2019	T: 30<br>C: 30	NR	T: 59. 67±14. 10<br>C: 58. 77±9. 48	T: 18/12<br>C: 19/11	NR	T: TA plus RT<br>C: RT	20 sessions, 4 w	30 min	LI10, LI11, LI14, LI15, SI9, SJ14, A-shi acupoint	VAS, FMA
Wen PX 2019	T: 30<br>C: 30	NR	T: 55.0±10.6<br>C: 53.2±9.2	T: 15/15<br>C: 16/14	NR	T: BAA plus RT<br>C: RT	21 sessions, 3 w	10 - 20 min	BP-LE6	VAS, FMA-U
Wen YC 2020	T: 34<br>C: 34	T: 2.1±0.3<br>C: 2.0±0.3	T: 65.8±9.3<br>C: 66.0±9.6	T: 20/14<br>C: 22/12	NR	T: EA plus RT<br>C: RT	28 sessions, 4 w	30 min	LI15, SJ14, Jianqian acupoint, SI9, A-shi acupoint, GB34, BP-LE6	VAS, FMA-U, MBI, ROM
Wu FC 2019	T: 60<br>C: 60	T: 41.9±10.8 d<br>C: 42.3±11.5 d	T: 58.39±8.96<br>C: 57.98±9.12	T: 36/24<br>C: 39/21	T: 25/35<br>C: 22/38	T: SA plus RT<br>C: RT	21 sessions, 3 w	2 h	2 acupoints zone (Ding zone, Dingqian zone)	VAS, FMA, MBI

Table 1 ***continues 3***

Study	Sample size	Time since stroke (m)	Age (mean±SD)	Sex (male/female)	Type of stroke (infarction/ hemorrhage)	Regimen	Treatment Duration	Needle retention duration	The selected acupoints	Main outcomes
Wu JY 2015	T: 40<br>C: 40	T: 56.2±17.3 d<br>C: 54.2±15.2 d	T: 69±8<br>C: 70±8	T: 21/19<br>C: 20/20	T: 24/16<br>C: 26/14	T: CAA plus RT<br>C: RT	24 sessions, 4 w	6 h	acupoints upper Four and Five	VAS, FMA-U
Xiao CH 2014	T: 22<br>C: 22	NR	T: 53.2±5.1<br>C: 52.8±4.8	T: 14/8<br>C: 13/9	T: 15/7<br>C: 14/8	T: WA plus RT<br>C: RT	14 sessions, 15 d	NR	LI4, LI10, LI11, LI15, SI9, SJ5, SJ14, Jianqian acupoint, A-shi acupoint, GB34	VAS, FMA-U, AE
Xu L 2011	T: 30<br>C: 30	T: 20 d～10<br>C: 20 d～3	NR	T: 18/12<br>C: 19/11	T: 20/10<br>C: 17/13	T: CAA plus RT<br>C: RT	20 sessions, 23 d	30 min	acupoints upper Four, Five and six	NR
Xun YJ 2019	T: 30<br>C: 30	T: 3.43±2.65<br>C: 3.47±2.80	T: 55.28±12.07<br>C: 54.06±11.21	T: 16/14<br>C: 18/12	T: 19/11<br>C: 21/9	T: EA plus RT<br>C: RT	20 sessions, 4 w	20 min	LI4, LI10, LI11, LI14, LI15, SJ5, SI9, SJ10, SJ14, A-shi acupoint	VAS, FMA-U, MBI
Yang RC 2018	T: 20<br>C: 20	T: 21. 00±3. 57 d<br>C: 21. 20±3. 14 d	T: 69.65±2.54<br>C: 69.70±2.83	T: 10/10<br>C: 11/9	T: 10/10<br>C: 11/9	T: EA plus RT<br>C: RT	20 sessions, 4 w	30 min	LI15, Jianhou acupoint, Jianqian acupoint	VAS, FMA-U, MBI
Zhang B 2012	T: 49<br>C: 51	NR	T: 54.3±10.2<br>C: 54.8±10.5	T: 31/18<br>C: 34/17	T: 26/23<br>C: 27/24	T: WA and EA plus RT<br>C: RT	60 sessions, 3 m	WA:30 min<br>EA: 15 min	WA: DU26, HT5, ST37, SP6, GB39, KI1<br>EA: LI4, LI11, A-shi acupoint	VAS
Zhang JK 2022	T: 40<br>C: 40	NR	T: 64.20±6.33<br>C: 63.57±6.81	T: 25/15<br>C: 22/18	T: 24/16<br>C: 22/18	T: EA plus RT<br>C: RT	20 sessions, 4 w	20 min	PC3, LU5, HT3, LI15, SJ14, GB21	VAS, FMA-U
Zhang Z 2014	T: 22<br>C: 20	NR	NR	NR	NR	T: EA plus RT<br>C: RT	12 w	30 min	LI4, LI10, LI11, LI15, SJ5, SI9, SI11, PC6	VAS, FMA-U, FIM
Zhang ZX 2012	T: 40<br>C: 40	10-102 (d)	56±16.65	45/35	26/54	T: BAA plus RT<br>C: RT	7 sessions, 7 d	10 - 20 min	BP-LE6	VAS, FMA-U
Zheng LQ 2020	T: 30<br>C: 30	T: 16.59±3.52 d<br>C: 15.96±3.42 d	T: 59.25±3.21<br>C: 59.12±3.13	T: 19/11<br>C: 20/10	T: 25/0<br>C:24/0	T: FA plus RT<br>C: RT	6 sessions, 14 d	30 sec	meridian-muscle nodes	VAS, FMA-U, MBI

Table 1 ***continues 4***

Study	Sample size	Time since stroke (m)	Age (mean±SD)	Sex (male/female)	Type of stroke (infarction/ hemorrhage)	Regimen	Treatment Duration	Needle retention duration	The selected acupoints	Main outcomes
Zhou GH 2002	T: 50<br>C: 50	NR	T: 53.8<br>C: 54.2	T: 32/18<br>C: 33/17	T: 40/10<br>C: 41/9	T: EA and BA plus RT<br>C: RT	28 sessions, 4 w	20 min	LI4, LI10, LI15, SJ14, SI9, LI11,SJ5	VAS

Notes. AA, abdominal acupuncture; AE, adverse event; BAA, Balancing acupuncture; BI, Barthel Index; BA, body acupuncture; CAA, Carpus-ankle Acupuncture; C, control group; CA, conventional acupoints; d, day; EA, electroacupuncture; FA, fire acupuncture; FMA, Fugl-Meyer Assessment Scale; FMA-U, Fugl-Meyer Assessment Scale for upper extremity; FPS, faces pain scale; m, month; MBI, modified Barthel Index; min, minutes; MPQ, McGi pain questionnaire; NR, not referred; NRS, numeric rating scale; RNN, relaxing needling at meridian-muscle nodes; RT, rehabilitation training; sec, second; SA, scalp acupuncture; T, treatment group; TA, traditional acupuncture; w, week; WA, warm acupuncture; VAS, visual analogue scale; ROM, range of motion of the affected shoulder; [#] the data is inappropriate and not included in meta-analysis.
